# Acceptability and Feasibility of a Socially Enhanced, Self-Guided, Positive Emotion Regulation Intervention for Caregivers of Individuals With Dementia: Pilot Intervention Study

**DOI:** 10.2196/46269

**Published:** 2023-09-06

**Authors:** Ian Kwok, Emily Gardiner Lattie, Dershung Yang, Amanda Summers, Veronika Grote, Paul Cotten, Judith Tedlie Moskowitz

**Affiliations:** 1 Northwestern University Feinberg School of Medicine Chicago, IL United States; 2 BrightOutcome Inc. Buffalo Grove, IL United States; 3 University of California San Francisco Osher Center for Integrative Medicine San Francisco, CA United States

**Keywords:** dementia, caregiving, eHealth, digital interventions, positive emotion, stress, coping

## Abstract

**Background:**

The responsibilities of being a primary caregiver for a loved one with dementia can produce significant stress for the caregiver, leading to deleterious outcomes for the caregiver’s physical and psychological health. Hence, researchers are developing eHealth interventions to provide support for caregivers. Members of our research team previously developed and tested a positive emotion regulation intervention that we delivered through videoconferencing, in which caregiver participants would meet one-on-one with a trained facilitator. Although proven effective, such delivery methods have limited scalability because they require significant resources in terms of cost and direct contact hours.

**Objective:**

This study aimed to conduct a pilot test of a socially enhanced, self-guided version of the positive emotion regulation intervention, Social Augmentation of Self-Guided Electronic Delivery of the Life Enhancing Activities for Family Caregivers (SAGE LEAF). Studies have shown that *social presence* or the perception of others in a virtual space is associated with enhanced learning and user satisfaction. Hence, the intervention leverages various social features (eg, discussion boards, podcasts, videos, user profiles, and social notifications) to foster a sense of *social presence* among participants and study team members.

**Methods:**

Usability, usefulness, feasibility, and acceptability data were collected from a pilot test in which participants (N=15) were given full access to the SAGE LEAF intervention over 6 weeks and completed preintervention and postintervention assessments (10/15, 67%). Preliminary outcome measures were also collected, with an understanding that no conclusions about efficacy could be made, because our pilot study did not have a control group and was not sufficiently powered.

**Results:**

The results suggest that SAGE LEAF is feasible, with participants viewing an average of 72% (SD 42%) of the total available intervention web pages. In addition, acceptability was found to be good, as demonstrated by participants’ willingness to recommend the SAGE LEAF program to a friend or other caregiver. Applying Pearson correlational analyses, we found moderate, positive correlation between social presence scores and participants’ willingness to recommend the program to others (*r*_9_=0.672; *P*=.03). We also found positive correlation between social presence scores and participants’ perceptions about the overall usefulness of the intervention (*r*_9_=0.773; *P*=.009). This suggests that participants’ sense of social presence may be important for the feasibility and acceptability of the program.

**Conclusions:**

In this pilot study, the SAGE LEAF intervention demonstrates potential for broad dissemination for dementia caregivers. We aim to incorporate participant feedback about how the social features may be improved in future iterations to enhance usability and to further bolster a sense of social connection among participants and study staff members. Next steps include partnering with dementia clinics and other caregiver-serving organizations across the United States to conduct a randomized controlled trial to evaluate the effectiveness of the intervention.

## Introduction

### Background

The persistent and progressive nature of dementia has deleterious effects not only for the care recipient but also for the caregiver, adversely affecting key aspects of psychosocial functioning [1,2]. The protracted burdens of caregiving may lead to elevated depression or anxiety [3], with increased duration and severity of symptoms heightening this risk among caregivers [4]. This has a tandem effect on outcomes for care recipients, where increased caregiving burden and stress results in diminished quality of care and quality of life for the patient [5].

To address the issue of caregiver burden, researchers are using new technologies to deliver resources to caregivers. One such intervention, Life Enhancing Activities for Family Caregivers, is a web-based positive emotion regulation intervention delivered through videoconferencing [6-8]. A randomized controlled trial of Life Enhancing Activities for Family Caregivers found that participation led to significant increases in positive emotion (Cohen *d*=0.58; *P*<.01) and positive aspects of caregiving (Cohen *d*=0.36; *P*<.01) and decreased symptoms of depression (Cohen *d*=−0.25; *P*=.02) and anxiety (Cohen *d*=−0.33; *P*<.01) compared with an emotion-reporting waitlist control group [6]. During this study, participants met one-on-one with a trained facilitator to learn the skills via videoconference. However, such delivery formats can be costly in terms of the time, effort, and logistics required to meet participants individually. To maximize the scalability of the intervention, there is a need for other delivery options that are more time efficient and cost efficient.

One such delivery format that has been widely adopted is the *self-guided* eHealth intervention, in which participants have access to the content on their own with minimal guidance from facilitators. However, although such interventions reduce barriers to participation, they are beset by high rates of attrition and poor adherence [9,10]. Parallel studies of other self-guided resources, namely Massive Open Online Courses, have found that the construct of *social presence*—or the perception of others being present in a web-based environment—can improve retention and engagement [11]. Features that enhance social presence include welcome messages, sharing participant profiles, and discussion boards [12]. Therefore, the application of such social features may improve engagement with eHealth and mobile health interventions.

### Objectives

Social features may be particularly beneficial for caregivers, who experience high levels of social isolation and loneliness compared with noncaregivers [13,14]. This study builds on data collected from focus groups and interviews conducted with providers and caregivers about the social features that might be most helpful for participants (Kwok, I, unpublished data, 2022). For example, focus group participants made suggestions to create podcast content, framing our reminder messages in an encouraging tone, and noted the potential challenges of creating a “buddy system” in which participants would be paired up. On the basis of these suggestions, we adapted the existing caregiver intervention and created Social Augmentation of Self-Guided Electronic Delivery of the Life Enhancing Activities for Family Caregivers (SAGE LEAF)—a socially enhanced, self-guided, web-based intervention for dementia caregivers that incorporated features that would enhance participants’ sense of social presence. In this study, our goal was to conduct a pilot study to determine the feasibility and acceptability of SAGE LEAF.

## Methods

### Participants and Study Procedures

A total of 15 dementia caregivers were recruited from social media advertisements and caregiver support groups and organizations, which often serve as the first point of contact for caregivers who are seeking resources and support. Although the age of onset and disease progression varies across different forms of dementia, caregivers experience a high level of emotional stress and burden, with similar support needs that include respite care, psychotherapy, and support groups.

Interested individuals were sent a screener survey to determine their eligibility based on the following inclusion criteria: (1) identify as the primary family caregiver of a person with dementia, (2) speak and read English, and (3) have access to high-speed internet. In our screener survey, we explained that a primary family caregiver is the caregiver who spends most time with the care recipient. However, we did not define what constitutes a family member to ensure that we were being inclusive of diverse familial arrangements. Caregivers were ineligible if they had participated in a previous version of the intervention. Study staff contacted eligible participants to explain the requirements of the study and seek informed consent. Once consented, participants were sent a link for the SAGE LEAF platform with instructions for creating their password. They were then able to access the intervention content, which included 8 positive emotion skills unlocked over the course of 6 weeks (description of the skills is given in the following section). During this period, they had access to all the social features of the platform.

Previous studies have found that 5 users may be sufficient to identify 80% of usability issues, with diminishing returns from additional testing [15]. Hence, the first 33% (5/15) of the participants were invited for a feedback interview at week 3 to identify any critical usability issues at the halfway point of the intervention that might significantly affect participation. This allowed them to provide feedback about the various features while they were still accessing the website. The subsequent 67% (10/15) of the participants were invited for a feedback interview at the end of the entire course. This would allow us to collect feedback from participants who had completed the whole intervention. However, the structure of the interview remained the same for all the participants (15/15, 100%).

### Intervention Content and Features

The SAGE LEAF intervention consists of 8 empirically validated positive emotion regulation skills that were delivered over 6 weeks. Each week, new content was unlocked for participants, with daily home practice exercises to reinforce the skills delivered that week. Similar to the previous versions of the intervention [6,16-19], the skills include noticing positive events, savoring, gratitude, mindful awareness, personal strengths, positive reappraisal, self-compassion, and attainable goals. SAGE LEAF is distinct from previous versions in that it has an emphasis on *social features* that are specifically designed based on the feedback we received from focus groups and interviews conducted with caregivers and clinicians who work with patients with dementia (Kwok, I, unpublished data, 2022). Such features include the design or functional elements of the platform that may enhance participants’ perceptions of social presence—the extent to which they sense the presence of others during their participation in the intervention. This includes sensing the presence of other caregiver participants and members of the study team. Textbox 1 shows a list of social features that were added to SAGE LEAF.

An example of how we incorporated the feedback from the focus groups and interviews (Kwok, I, unpublished data, 2022) was adopting a more encouraging tone in our automated support features. For instance, if a participant did not log in for a week, an encouraging message was sent to them through their preferred communication method (email or SMS text message). Other social features that we created based on feedback include the multimedia elements of the intervention. For example, in addition to an introduction video shown at the beginning of the study, the study team members recorded a skill-building video at the beginning of each lesson, briefly describing the skill and how it may be potentially helpful for caregivers. Furthermore, we recorded podcasts at the end of each lesson, which provides caregivers with optional content that they can review at their own schedule. The podcasts comprised interviews with study team members explaining how caregivers could overcome some of the challenges with applying the skills in everyday life and how to enhance their practice of the skills. On the basis of the feedback received, we also added more fields for the user profiles, so that participants could choose to share various aspects of their caregiving experience, for example, the relationship between the caregiver and recipient and some of the challenges and positive experiences that they may have had as a caregiver.

**A list of social features in the Social Augmentation of Self-Guided Electronic Delivery of the Life Enhancing Activities for Family Caregivers intervention.**

**Videos**

- When participants log into the website for the first time, they are shown a short welcome video in which a study team member provides an overview of the study. At the beginning of each skill-building lesson, participants can view a short video in which study team members introduce the skill and provide key takeaways about the topic. This also allows participants to see the team members behind the study, which lends a personal touch to their learning.

**Podcasts**

- At the end of the skill-building lessons for each week, participants have the option to listen to a short audio recording of staff members discussing how to apply the skills for the week in an informal, conversational format. This enhances the sense that team members have unique, personal perspectives about the skills.

**Library**

- This is a repository for all unlocked videos, podcasts, and mindfulness meditation recordings that participants can access.

**Discussion board**

- Participants have access to the moderated discussion board, which is organized according to the different positive emotion skills and is moderated by a study team member. This encourages social interaction around the content being delivered. Participants also have the option to post their home practice activities for each skill on the discussion board. Participants can respond to others’ posts or send a “like” or “thank you.”

**Community tab**

- Participants are able to view the profiles of other participants who are on the web at the same time and the profiles of all enrolled participants. This enhances the perception that there are other caregivers going through the study at the same time.

**Emotions chart**

- Participants’ daily emotion survey data are displayed in a graph representing their positive and negative emotions over the past week. Although this feature does not allow for social interaction, it enhances the perception that participants’ inputs are being reflected in the feedback provided by the system or study team.

**Social notifications**

- Participants receive notifications on their dashboard, via email, or via SMS text message if others responded to their posts or sent “like” or “thank you.” These prompts call attention to social interactions that are occurring around caregivers’ posts and encourage continued dialogue or interaction.

**Automated reminders**

- On the basis of their preferences, participants receive encouraging email or SMS text message reminders to complete their daily home practice and emotion surveys or if they do not register their password or log into the website for several days during the intervention. This may enhance caregivers’ perceptions that the study team is responsive to them and that their continued participation is important.

**User profiles**

- Participants have their own user profile page where they can select their own avatar or provide answers to questions around their caregiving experience or circumstance; for example, “What are some challenges that you’ve experienced as a caregiver?” Participants are able to view each other’s profiles through the discussion board or community tab, which reduces the anonymity of participating in the intervention and allows participants to learn more about other caregivers participating in the study.

**Control panel**

- Within their user profiles, participants have access to settings that allow them to select whether they would like to receive notifications and reminders through email or SMS text message. This feature is not inherently social but provides participants with control over features that may have a social component.

### Study Evaluation and Measures

All participants completed a 45- to 60-minute phone interview to provide feedback about their experience of using the platform, with a focus on the social features being implemented. Participants dialed into an audio-only Zoom (Zoom Video Communications) session that was recorded. The interview followed a semistructured guide and was conducted with a trained facilitator who has experience with conducting focus groups and interviews (Kwok, I, unpublished data, 2022). The questions evaluated participants’ (1) use of the social features, (2) facilitators of or barriers to using the social features, and (3) recommendations for future improvements for each of the social features: for example, “One of the features of the website was the discussion board. Were you able to use the discussion boards? How often did you use them? If not, what kept you from using them? What are some aspects of the discussion boards that you found helpful? What are some aspects of the discussion boards that you didn’t find helpful? What do you think we could do to encourage people to participate in the discussion boards? How might the discussion boards be improved?” These questions were repeated for each social feature.

### Qualitative Analyses

All interviews were recorded and automatically transcribed using Zoom’s cloud recording feature. Content analysis was conducted by categorizing user feedback as positive, negative, or implementation suggestions corresponding to each social feature. In addition, we noted any issues that participants might be having with the other aspects of using the SAGE LEAF website.

### Measures

Participants completed the surveys at baseline and after the intervention at week 8. We added a 1-week buffer before and after the 6-week intervention, so that we could ensure that participants had fully completed the study before sending out the postintervention surveys. The postintervention survey included our primary measures that assessed the (1) usability, (2) usefulness, (3) feasibility, and (4) acceptability of the SAGE LEAF platform. Furthermore, we measured the participants’ perceptions of others on the SAGE LEAF platform by using an adapted version of the original Social Presence Scale [20]. Although the study was not designed to determine the efficacy of the intervention, we included measures to examine pre-post changes in measures of caregiving burden and psychological well-being. A list of measures is shown in Table 1.

**Table 1:** List of measures and constructs with descriptions.

Measures and constructs			Description and examples
**Primary measures and constructs**			
	System Usability Scale	This is a 10-item measure widely adopted in user experience studies to determine system usability across diverse technologies.	
	Feature-specific usefulness	We adapted the Perceived System Usefulness scale to rate the perceived usefulness of the individual social features of the website (eg, “Using the discussion board would be helpful for my learning,” with a 5-point Likert scale ranging from 1 [strongly disagree] to 5 [strongly agree]).	
	System-wide usefulness	This is a measure that was tailored to rate the perceived usefulness of the SAGE LEAF^a^ website as a whole (eg, “The system was useful in helping me learn the positive emotion skills,” with a 5-point Likert ranging scale from 1 [strongly disagree] to 5 [strongly agree]). This system-level evaluation of usefulness is often adopted in health care technology studies [21].	
	Feasibility	Adherence was assessed as the proportion of pages viewed out of the total possible pages in the intervention, and retention was assessed as the percentage of participants who completed the postintervention assessment.	
	Acceptability	We asked participants’ willingness to recommend the SAGE LEAF program to a friend or dementia caregiver using an 11-point Likert scale ranging from 0 (definitely no) to 10 (definitely yes).	
	Social Presence Scale	An adapted version of the original Social Presence Scale [20], which includes 14 items relating to social presence on a 5-point Likert scale ranging from 1 (strongly disagree) to 5 (strongly agree). Items include “The SAGE LEAF website is an excellent place for social interaction” and “I felt comfortable participating in discussions on the SAGE LEAF discussion boards.” We added an additional item to account for perceptions of social presence of other participants and study staff separately. The Social Presence Scale has been shown to have a high level of reliability (Cronbach α=.88) and has been adapted to evaluate a broad range of web-based communities [11].	
	Social connection	This comprises 4 items to capture a general sense of social connection to others, where participants reported the extent to which they felt isolated or connected to others, had a lot in common with others, or had people they can relate to. These items were rated on a 7-point Likert scale ranging from 1 (strongly disagree) to 7 (strongly agree).	
**Preliminary outcomes**			
	Zarit Burden Interview	This is a 22-item measure that evaluates caregiving burden [22]: for example, “Do you feel that because of the time you spend with your relative that you don’t have enough time for yourself?”	
	OCBS^b^	This is a 15-item measure that assesses the (1) perceived amount of time spent (OCBS-Time) or (2) difficulty of tasks (OCBS-Difficulty) relating to various caregiving activities [23]: for example, “Emotional support, ‘being there’ for the patient; Please select how much time you spend on this task and Please select how difficult it is to do this task.”	
	Positive Aspects of Caregiving Scale	This is an 11-item measure that evaluates caregivers’ outlook about life and self-affirmations [24]: for example, “Providing help to (Care Recipient) has made me feel needed, appreciated, important, etc.”	
	Caregiver Reaction Scale	This is a measure that evaluates the socioemotional effects of caregiving, for which the 2 domains of role captivity and overload relate to caregiving burden [25]: for example, “How much does each statement describe your thoughts about caregiving? Wish you could just run away. Feel stressed by your relative’s illness and needs.”	
	Differential Emotions Scale	Positive and negative affect was assessed as the extent to which participants may or may not have experienced various emotions (eg, peaceful, interested, and guilty) over the past week [7].	
	Perceived Stress Scale	This is a 10-item measure that assesses the extent to which participants evaluate their circumstances to be uncontrollable, unpredictable, or overloaded [26]: for example, “In the last month, how often have you felt that you were on top of things?”	
	Satisfaction With Life Scale	This is a widely administered 5-item measure of global evaluations of life satisfaction [27]: for example, “In most ways my life is close to ideal.”	
	Patient-Reported Outcomes Measurement Information System	This is a toolbox of instruments developed by the National Institute of Health [28], which measures broad aspects of psychosocial functioning. We included the instruments for positive affect, social isolation, anxiety, depression, meaning and purpose, and sleep disturbance, which represent salient aspects of a caregiver’s psychosocial functioning.	

^a^SAGE LEAF: Social Augmentation of Self-Guided Electronic Delivery of the Life Enhancing Activities for Family Caregivers.

^b^OCBS: Oberst Caregiving Burden Scale.

### Statistical Analyses

We calculated the medians and IQR for primary measures that assessed the (1) usability, (2) usefulness, (3) feasibility, and (4) acceptability of the SAGE LEAF platform. In addition, we calculated supplementary user metrics based on data collected by the platform (eg, percentage of home practice activities completed and percentage of videos watched) that reflect various aspects of engagement. We calculated the means and SDs for the Social Presence Scale and applied them as a correlate with measures of usability, usefulness, feasibility, and acceptability. For measures related to preliminary outcomes, we performed paired, 1-tailed *t* tests on the data collected during the baseline and postintervention assessments to examine changes in means. Analyses were conducted using Excel (Microsoft Corporation) and R Studio (Posit).

### Ethics Approval

The Northwestern University Institutional Review Board reviewed and approved the protocol for this study (Reference number: STU00215548).

## Results

### Study Recruitment, Enrollment, and Retention

Participants were recruited from a group of individuals who responded to social media advertisements for another caregiver study but were not eligible (3/15, 20%) and from dementia caregiver support groups (12/15, 80%). A total of 25 participants were screened, of which 10 (40%) were excluded owing to the following reasons: the care recipient did not have a diagnosis of dementia (4/25, 16%), they were no longer interested in participating in the study (2/25, 8%), and they dropped out before enrollment (4/25, 16%). Eligible participants provided web-based informed consent; completed a baseline assessment; and were subsequently sent an email with instructions about how to set up their account on the SAGE LEAF website, which would give them access to the 6-week program. Upon the completion of the SAGE LEAF course, they completed a follow-up assessment. In addition, all participants (15/15, 100%) completed a feedback interview, with (1) the first 33% (5/15) of the participants scheduled to complete the interview at week 3 to determine if there were significant usability issues early in the progression through the program, and (2) the next 67% (10/15) of the participants scheduled to complete the same feedback interview after they had completed the intervention. Initially, the qualitative feedback from the first 33% (5/15) of the participants was examined separately. However, given that there were no significant usability issues identified among them and that the subsequent 67% (10/15) of the participants completed exactly the same version of the intervention, the qualitative feedback and quantitative data were combined for analysis. The baseline characteristics of participants are shown in Table 2.

**Table 2:** Baseline characteristics (N=15).

Participant characteristics		Values
**Gender, n (%)**		
	Female	11 (73)
	Male	4 (27)
Age (years), mean (SD)		62.80 (11.31)
**Race and ethnicity, n (%)**		
	Hispanic or Latinx and White	2 (13)
	Non-Hispanic and White	13 (87)
**Relationship with patient, n (%)**		
	Spouse	13 (87)
	Other family member	2 (13)
Duration of caregiving (years), mean (SD)		4.27 (2.94)
**Diagnosis, n (%)**		
	Frontotemporal dementia	11 (73)
	Uncategorized dementia	4 (27)

### Feasibility, Acceptability, Usability, and Usefulness

User metrics and measures for feasibility, acceptability, usability, and usefulness were collected upon completion of the intervention. Feasibility of the SAGE LEAF intervention was determined as the average percentage of number of web pages viewed out of the total 139 pages available in the intervention. These data were collected by the SAGE LEAF website. Participants demonstrated good adherence, completing an average of 72% (SD 42%; 100/139 pages) of the pages of the intervention.

Other measures for acceptability, usability, and usefulness were collected using the postintervention survey. As these data were not normally distributed owing to a relatively small number of participants, we calculated the medians and IQRs for these variables. Acceptability was evaluated to be good, with participants rating their willingness to recommend the SAGE LEAF program to a friend (median 8; IQR 6-8) or dementia caregiver (median 8; IQR 6.25-9.5), with 1 indicating *definitely not* and 10 indicating *definitely yes*. Usability was assessed using the System Usability Scale, with a median score of 73.75 (IQR 57.5-85.63) out of a total of 100, which can be interpreted as “good” from a usability perspective [29]. In terms of *feature-specific* usefulness, on a scale of 1 to 5, participants rated the discussion board with a median score of 3 (IQR 2-3), email reminders with a median score of 3 (IQR 2-4), user profiles with a median score of 2 (IQR 2-3), videos with a median score of 3 (IQR 3-3.75), podcasts and audio recordings with a median score of 3.50 (IQR 3-4), and social notifications (ie, alerts about likes and comments) with a median score of 3 (IQR 2-3). The ratings suggest that the podcast and audio recordings were determined to be the most useful feature. In terms of *system-wide* usefulness, participants rated SAGE LEAF with a median score of 3.70 (IQR 3-4.75) on a scale of 1 to 5.

Regarding social presence, participants rated the SAGE LEAF program with a median score of 3 (IQR 2.66-3.33) on a range of 1 to 5. Studies of the Social Presence Scale have not yet established benchmarks for what constitutes optimal levels for web-based platforms. However, past studies demonstrate strong associations between social presence scores and student’s satisfaction and learning, in the context of e-learning platforms [11]. Hence, we performed exploratory Pearson correlational analyses with social presence scores as a correlate with feasibility, acceptability, usability, and usefulness ratings. Our analyses found moderate, positive correlation between social presence scores and participants’ willingness to recommend the program to others (ie, friends and other caregivers; *r*_9_=0.672; *P*=.03). We also found positive correlation between social presence scores and participants’ perceptions about the overall usefulness of the intervention (*r*_9_=0.773; *P*=.009). We found no statistically significant associations with other feasibility, usability, and usefulness measures. Table 3 shows the results of these measures.

**Table 3:** Usability, usefulness, feasibility, and acceptability measures.

Metrics and measures			Values
**SAGE LEAF^a^ website metrics (N=15), mean (SD)**			
	Total number of skills content pages reviewed		106.73 (62.50)
	Total skills content completed (%)		72 (42)
**User measures (n=10; scale ranging from 1 to 5 or as indicated), median (IQR)**			
	System Usability Scale (total=100)		73.75 (57.5-85.63)
	**Feature-specific usefulness**		
		Discussion board	3 (2-3)
		Email reminders	3 (2-4)
		User profiles	2 (2-3)
		Videos	3 (3-3.75)
		Podcasts and audio recordings	3.50 (3-4)
		Social notifications	3 (2-3)
	System-wide usefulness		3.33 (3-4.75)
	Social Presence Scale		3 (2.66-3.33)
**Acceptability (n=10; scale ranging from 1 to 10), median (IQR)**			
	Would recommend to a friend		8 (6-8)
	Would recommend to a caregiver		8 (6.25-9.5)

^a^SAGE LEAF: Social Augmentation of Self-Guided Electronic Delivery of the Life Enhancing Activities for Family Caregivers.

### Preliminary Outcomes

Of the 15 participants, 5 (33%) were lost to follow-up. The remaining 67% (10/15) of the participants completed both the baseline and postintervention surveys, which included measures relating to preliminary outcomes (Table 4). One-tailed paired *t* tests demonstrated a statistically significant (1) decrease in negative affect from baseline (*M*=1.71, SD 0.78) to after the intervention (*M*=1.34, SD 0.72; *t*_9_=−2.49, *P*=.03); (2) decrease in perceived stress from baseline (*M*=9.00, SD 1.63) to after the intervention (*M*=7.50, SD 1.78; *t*_9_=−2.29, *P*=.05); and (3) decrease in anxiety from baseline (*M*=61.42, SD 5.00) to after the intervention (*M*=57.45, SD 7.96; *t*_9_=−2.52, *P*=.03); we also found a statistically significant (4) increase in meaning and purpose from baseline (*M*=43.56, SD 7.33) to after the intervention (*M*=47.19, SD 5.63; *t*_9_=2.60, *P*=.03). No other statistically significant changes were found between the baseline and postintervention findings. However, no definitive conclusions about efficacy can be made, because our pilot study did not have a control group and was not sufficiently powered.

**Table 4:** Preliminary outcome measures (n=10).

Measures			Baseline score, mean (SD)		Postintervention score, mean (SD)		Difference, mean (SD)		*t* test (*df*)^a^		*P* value		Cohen *d*
**Primary measures**													
	Zarit Burden Interview	16.40 (4.17)		15.67 (3.54)		−0.89 (3.10)		−0.86 (9)		.41		−0.29	
	OCBS^b^—time	3.07 (0.75)		3.07 (0.75)		−0.01 (0.27)		−0.06 (9)		.95		−0.02	
	OCBS—difficulty	2.71 (0.78)		2.77 (0.82)		0.05 (0.41)		0.40 (9)		.70		0.13	
	Positive Aspects of Caregiving Scale	22.23 (8.91)		22.23 (9.27)		0.09 (6.75)		0.03 (9)		.98		0.01	
	CRS^c^—role captivity	2.58 (0.64)		2.55 (0.70)		−0.03 (0.55)		−0.14 (9)		.89		−0.05	
	CRS—overload	2.92 (0.69)		2.65 (0.83)		−0.27 (0.74)		−1.14 (9)		.28		−0.36	
	Caregiving Mastery Subscale	2.55 (0.71)		2.66 (0.45)		0.11 (0.59)		0.57 (9)		.58		0.18	
**Other measures**													
	Positive affect (Daily Emotion Survey)	1.65 (0.68)		1.82 (0.68)		0.18 (0.46)		1.20 (9)		.26		0.38	
	Negative affect (Daily Emotion Survey)	1.71 (0.78)		1.34 (0.72)		−0.38 (0.48)		−2.49 (9)		.03^d^		−0.79	
	Cohen Perceived Stress Scale	9 (1.63)		7.50 (1.78)		−1.50 (2.07)		−2.29 (9)		.05^d^		−0.73	
	Satisfaction with Life Scale	16.60 (5.19)		17.80 (5.69)		1.20 (3.74)		1.02 (9)		.34		0.32	
	PROMIS^e^—social isolation	53.30 (6.93)		51.27 (5.73)		−2.03 (4.75)		−1.35 (9)		.21		−0.43	
	PROMIS—anxiety	61.42 (5)		57.45 (7.96)		−3.97 (4.98)		−2.52 (9)		.03^d^		−0.80	
	PROMIS—depression	59.59 (4.44)		57.96 (5.05)		−1.63 (4.30)		−1.20 (9)		.26		−0.38	
	PROMIS—meaning and purpose	43.56 (7.33)		47.19 (5.63)		3.63 (4.41)		2.60 (9)		.03^d^		0.82	
	PROMIS—sleep disturbance	51.65 (5.22)		52.73 (7.27)		1.08 (4.45)		0.77 (9)		.46		0.24	
	NIH^f^—positive affect	39.20 (11.45)		42.20 (10.71)		3 (6.18)		1.53 (9)		.16		0.49	

^a^One-tailed *t* test.

^b^OCBS: Oberst Caregiving Burden Scale.

^c^CRS: Caregiver Reaction Scale.

^d^Statistically significant *P* values; *P*≤.05.

^e^PROMIS: Patient-Reported Outcomes Measurement Information System.

^f^NIH: National Institute of Health.

### Feedback Interviews

#### Initial Feedback About Social Features

The first 33% (5/15) of the feedback interviews (conducted midway through the 6-week program) revealed that participants’ use and understanding of how the social features worked varied greatly. For example, some participants noticed the social notifications and the number of “post views” on the discussion board, visited other members’ profiles, and commented or liked other users’ posts. Other participants did not realize that they could customize their own user profile or view other users using the community tab. One of the initial user test participants suggested that we should provide more guidance about the various features of the website. Hence, we recorded a brief tutorial video about how to use the SAGE LEAF website, which was then included as a link in an email to all participants, highlighting the different social features of the website:

Yes, I did see them [social notifications]...and then I realized that you know someone was actually reading...because i’ve done that to other people’s posts...it’s like being on Facebook, but it’s good because it makes it makes you feel you know someone is actually realizing what you’re going through and thanking you for sharing that.

Participant 02

No other usability issues were identified from the feedback. Hence, we did not make any other additional modifications to the website or intervention following the first 33% (5/15) of the user tests. As all participants (15/15, 100%) received the same version of the intervention, the subsequent feedback was aggregated and organized according to the various social features.

#### Podcast and Video Content

Generally, there was positive feedback about the video and podcast content. Participants liked being able to learn more about the skills from the perspective of team members while being able to see or hear the people behind the study:

It was nice to get that personal touch I did like that...You know the fact that it was you know people that are working as part of the study...it shows that there’s a...sense of ownership...

Participant 01

Participants described how the videos were able to enhance their awareness about the study staff involved in running the intervention. In addition, they praised the format of the videos in terms of being able to communicate key takeaways in a short amount of time:

...Hearing other people’s voices or we’re seeing their faces, I think that made it feel more like being part of like a class or something a little more social aspect to it than just the reading and answering the questions.

Participant 08

However, there were suggestions about how this content could be improved. For example, a participant mentioned that the combined time required to complete the video, didactic content, and podcasts in one sitting might be a lot for caregivers with busy schedules. Although the videos and podcasts were optional and participants could choose to return to this content at any point, this participant suggested that we send out optional content such as the podcasts sometime later in the week to reinforce their learning.

In addition, 13% (2/15) of the participants mentioned that they felt that some of the information provided in the videos overlapped with that in the lessons. They offered suggestions to include case studies and testimonials featuring other caregivers in the videos or podcasts, which would complement the lessons. They also suggested including examples of how the skills could be used in more stressful caregiving situations:

Case studies would be good, you know where you could see maybe how they’ve [positive emotion skills] helped other people...maybe have some people who would be willing to give things from an actual caregiver perspective...Like if you’re talking about you know positive reinforcement or whatever, if someone could say, well, I had this experience, and this is how it helped me.

Participant 11

#### Discussion Board

Several participants expressed positive feedback about the discussion boards, which provided them with a medium to share their feelings or experiences with using the positive emotion skills:

I thought, “Okay we’ll give it [discussion board] a try.”...and it was nice getting the feedback, “Oh, you know I understand where you are.”...I guess it was kind of nice to read through some of them and realize that I’m not the only one dealing with with all of this, which I knew before, but it’s nice to be reminded of that...

Participant 06

Furthermore, most participants noticed the social notification feature, which provided them with a prompt when other users liked or replied to their posts on the discussion board. However, other participants did not find the discussion board as helpful because they noticed that participants were primarily posting answers to their home practice activities instead of responding to each other’s posts:

I look to see what other people were putting down and out of all the people who were on there, I think, maybe, only three of us actually put information down about ourselves. And the rest of the people I think chose not to reveal anything about themselves. So there really wasn’t any bonding, so to speak.

Participant 13

A participant suggested promoting “popular” or recent posts on the home page as a way of encouraging engagement among caregivers by highlighting content that may be interesting to them. Additional feedback indicated that there was interest in broadening the scope of the discussion board to include more general topics. This would allow participants to foster a sense of community and social connection:

I wish to keep the skill-focused discussion, which is great...people trying to understand the skills and that’s important...and then have the open-ended conversation where people are talking about day to day life in their situation...hopefully they’re bringing their own understanding of the skills to that situation.

Participant 03

#### Automated Reminders

The pilot test revealed that most participants were completing their daily emotion surveys and home practice activities regularly. This may be attributed to the automated reminders that we programmed for when new lessons were unlocked or when participants did not register their accounts or log into the platform for several days. When asked about whether it might be helpful to increase the frequency of reminders, a participant suggested that the frequency might become very high. Another participant suggested that the reminders sounded “generic” and could be personalized by study staff members:

And it just occurred to me another thing that the emails were generic...there was no personality there. Since we’re meeting people in the course of the video program, it might be something where you can take advantage of these people and use them as the voice and face of some of these emails. This is so, and so, and you know I spoke to you this week about that and just want to...just know that we’re all here to support you...So its not just another email.

Participant 05

A participant suggested that having the reminders worded differently each day with a thoughtful message may encourage future participants to read these emails and to log into the platform:

I think, for me it would be just a simple reminder, but it wouldn’t say the same thing, every day, I would get an email saying, “Oh it’s Earth Day...and don’t forget to do your daily check in.”...So it’s not the same thing every day, because I’m sure that most people are like me, and I get so many emails every day.

Participant 06

#### User Profiles and Control Panel

Although 33% (5/15) of the participants completed their user profiles, others were not aware that they could customize their user profile by selecting an avatar, providing answers to questions about their caregiving circumstance, or customizing their preferences to receive notifications via email or SMS text message. On the basis of preliminary feedback, we had created a tutorial video explaining how the various social features worked and sent it to pilot test participants via an email link. However, participants’ lack of awareness about the user profile feature suggests that they may not have watched this video. In addition, some participants were not aware about the community tab, which showed the profiles of all participants in the study. Hence, a participant suggested that the study should encourage users to complete their profiles and select their control panel preferences before the beginning of the lessons:

I think when we initially sign in that’s usually like the best time where you can kind of be like, “Oh, do you want to receive text message notifications?” And you can kind of like set it all up at the very start.

Participant 08

#### Emotions Feedback Chart

Most participants had accessed the emotions chart tab on the landing page, and several found this feature to be helpful because this feedback enhanced their awareness about the emotions that they were experiencing. However, similar to the community tab that was located on the landing page, some users were not aware that the website included this feature. This feedback reiterates the need to explain how the social features work or how they may be beneficial for participants. For example, when participants are completing their daily emotion surveys, we could remind them that their data will be reflected in the emotions chart as a way of encouraging them to complete the surveys:

It was interesting...and kind of reflect back on, “Oh well, you know I wasn’t having a good day that day,” or, “That day went pretty well,” so it was just kind of interesting to see about how many days are bad or how many days are good.

Participant 11

#### Other Feedback

We also asked participants for suggestions about other social features that might encourage social connection. A participant suggested the use of chat rooms, which would allow future users to connect in real time. Another participant discussed how it might be helpful to have caregivers join the study in small groups and have their profiles shared among each other before joining the study:

If there was five or six people or whatever...and that we would have the opportunity to kind of get to know each other and our backgrounds and to make our sharing about our situations and our challenges more meaningful...I’d be more inclined to do that, rather than just all these people up there...I don’t know is there 2000 of them, or eight of them, or what you know...

Participant 06

The importance of the onboarding process was reiterated by another participant who suggested that future participants may be motivated to use the various social features if they were informed about how they could use them as tools for fostering social connection.

Other participants suggested that future users would benefit from having additional context about how and why the features might be beneficial for them in various ways. This could be included in reminders or brief instructions on the SAGE LEAF website, which would encourage the use of the social features:

Give them like a reminder, like you can even put a little note in the “Community” thing like, “Hey don’t forget to fill out your profile and you can look at other people’s profile,” or you know what I mean just a little reminder like that.

Participant 05

Taken together, this feedback suggests that providing timely reminders about how the social features work and how they might be helpful may encourage future participants to use them. A summary of the feedback collected about each social feature and potential implementation ideas is presented in Table 5.

**Table 5:** Summary of feedback and potential implementation ideas.

Feature	Summary of feedback	Future implementation ideas
Videos and podcasts	Participants enjoyed the multimedia content, and these features allowed them to hear from the study team members involved in the study. However, some participants found the content as repetitive and wanted to hear from other caregivers.	Including testimonials and case studies from other caregivers in the video and podcast content
Discussion board	Participants generally liked the discussion boards but found that other participants were mostly posting their home practice activities instead of engaging with each other.	Promoting recent or popular posts Including additional discussion boards that are more open ended and unrelated to the skills
Automated reminders	Participants suggested that the automated reminders seemed “generic.” They also indicated that sending daily reminders might be very overwhelming for caregivers.	Including thoughtful or customized messages in the reminders Maintaining the existing frequency of reminders instead of daily reminders
User profiles and control panel	In total, 33% (5/15) of the participants completed their profile information, with several other participants indicating that they did not know about the user profile and control panel features.	Incorporating the completion of user profiles during the onboarding process
Emotions feedback chart	Some participants were not aware about this feature.	Reminding participants that their daily emotion survey data will be displayed in the emotions feedback chart when they are completing the survey
Other feedback	Participants’ understanding of how the features worked varied greatly.	Creating tutorial slides or videos that participants access during the onboarding process, which would explain how the features work and why they might be helpful for caregivers Adding brief instructions for the features, explaining how they work and why they might be helpful for caregivers
Other feedback	Participants suggested implementing features that could further enhance a sense of connection among caregivers.	Including chatrooms Recruiting small cohorts of caregivers

## Discussion

### Principal Findings

In this study, we adapted an existing intervention that has been previously tested with caregivers by incorporating social features into the design of the platform, which include podcasts and videos from the study team, multimedia library, discussion board, community tab, emotion feedback chart, social notifications, automated reminders, and user profiles. We then conducted a pilot test to determine its feasibility and acceptability. Participants’ ratings showed that the SAGE LEAF intervention demonstrated a high level of feasibility and acceptability.

Participants provided feedback about how to improve the social features in a way that would foster great social presence. Participants liked the format and content of the videos and podcasts, which enhanced their perception of the presence of study team members involved in the intervention. However, these could be improved by featuring caregivers through case studies or testimonials or discussing how the skills could be used in acutely stressful situations. The interviews revealed that most participants used the discussion board to post their home practice answers. However, there was less interaction among participants in response to each other’s posts. A suggestion was to highlight new or relevant posts that might entice users to interact with each other. The feedback indicates that the participants’ experience with using the discussion board may be enhanced if they had had more understanding about how other related social features work, including the user profiles, social notifications, automated reminders, and community tab—which may have synergies in enhancing the perception that there are other caregivers using the platform. Several participants also liked the emotions feedback chart because it provided feedback about their emotion data, but some participants were not aware about this feature.

In general, participants suggested that it would be important to explain why the features might be beneficial for caregivers early in the intervention. Hence, future versions of SAGE LEAF may incorporate this information in a tutorial video or slides during the registration process or encourage participants to select an avatar and complete their profile when they first log into the website, so that it can help them connect with other caregivers. Hence, we hope to enhance the onboarding process in future versions of SAGE LEAF, so that it sets up participants for successful social interactions during their time in the program. Given the web-based, self-guided format of the intervention, this initial setup process may be particularly crucial owing to the lack of direct contact between participants and study staff members. Other implementation suggestions from participants include enrolling small cohorts of participants and sharing relevant aspects of their profiles with the group. This may help establish a more personal context for interacting over the web and foster a strong sense of social connection.

### Further Studies and Implications

Building on these early findings, future studies may include a randomized controlled trial of the SAGE LEAF intervention, in which a large pool of participants may be assigned to different combinations of the social features through a factorial design. A previous iteration of the intervention for individuals with depression showed that the combination of internet-based rewards and brief facilitator contact resulted in participants completing more of the intervention compared with those who only had access to only 1 feature [30]. A factorial design may be helpful in understanding which SAGE LEAF social features may have unique synergies for dementia caregivers. For example, for SAGE LEAF, the combination of user profiles with the discussion board may allow participants to learn more about the other participants who they are communicating with, thereby enhancing a sense of social presence. It is also plausible that other social feature combinations may work against each other such that a lot of social features may be burdensome for participants because our feedback interviews revealed that receiving daily reminders or completing the videos, skill lessons, and podcasts in one sitting is difficult for many caregivers. In addition, participants might feel pressured to share information or compete in internet-based challenges if all the activities have a social component. Hence, this future study will be helpful in teasing apart how such social feature combinations may influence outcomes.

Moving forward, once shown to be efficacious, the program may be broadly disseminated through dementia clinics across the country. For example, a local clinic may implement the SAGE LEAF intervention by enrolling a cohort of caregivers living in the same geographic area, which may complement their ongoing support group programming. Furthermore, dementia clinics may use the SAGE LEAF platform to connect participants who provide care for recipients with more uncommon forms of dementia. The web-based format of SAGE LEAF could facilitate participation across clinics in different geographic locations, allowing caregivers to connect with others who might share their unique circumstances.

### Strengths and Limitations

One of the strengths of this study is that the social features that we incorporated were based on a previous study in which we collected qualitative feedback from clinical providers and caregivers about which features would be most helpful for caregivers (Kwok, I, unpublished data, 2022). We also performed a pilot test in which participants were given access to a fully functional version of the SAGE LEAF website, which would allow them to engage in these social features as if they were actual users with other participants enrolled at the same time. Typically, user experience evaluation studies are conducted through brief *momentary evaluations* or *episode tests*, with a trained facilitator having participants go through various tasks, collecting participants’ comments in real time, and recording the time that it takes to complete these tasks and their completion rates [31]. Instead, the pilot test allowed participants to evaluate the usefulness of our social features in a more naturalistic way and help us identify any significant usability issues.

A limitation of our study is our small sample size (N=15), which limits the generalizability of the findings. The small sample size allowed for a detailed and in-depth examination of usability and feasibility concerns through a mix of quantitative and qualitative methods. However, a large cohort of participants may have revealed more insights about the usability of the social features.

Another limitation of the study is that our sample lacked diversity in the type of diagnosis of dementia, with most participants being caregivers of a family member with frontotemporal dementia. There are >100 known forms of dementia, with Alzheimer disease being the most common diagnosis [32], and there is significant variation in the presentation of these diagnoses. In terms of differences in the presentation of dementia, Alzheimer disease affects individuals at an older age compared with frontotemporal dementia. In addition, Alzheimer disease results primarily in memory impairments as compared with the significant changes in language skills and personality exhibited by individuals with frontotemporal dementia. However, although care needs may differ, caregiving burden remains high across diagnoses, and the positive emotion skills may be helpful in managing the stress of caregiving. Therefore, the insights collected about feasibility and acceptability from this pilot study are likely to be generalized across diagnoses. Future studies may involve comparing cohorts of participants with care recipients with similar diagnoses and confirming that the intervention is effective across diagnoses. These cohorts may also be recruited based on the relationship between the caregiver and care recipient (eg, children caring for their parent or parent-in-law vs caregivers who are partners or spouses), which may enhance a sense of social connection among participants who share similar caregiving circumstances.

Furthermore, our sample lacked ethnic diversity, with non-Hispanic, White participants comprising 87% (13/15) of all participants. This warrants recognition because racial and ethnic minority communities are disproportionately affected by dementia. For example, dementia is approximately 50% more prevalent among Mexican American older adults [33] and Alzheimer disease is twice as prevalent among Black older adults than among their White counterparts [34]. In addition, as internet access varies across ethnic groups, our sample may have been more familiar with using the internet and email to participate in the study. Future collaboration with community groups relating to older adult services, family services, faith-based groups, and so on may help with outreach for underserved caregivers [35]. For large-scale evaluations of SAGE LEAF, relevant recruitment strategies may include tailoring our efforts according to geographic location, providing monetary incentives for engaging in more specific components of the study (eg, completion of each lesson and home practice activity), affording great flexibility in how we contact participants, and providing more extensive recruitment training [36-38].

### Conclusions

Through our pilot test, we found the SAGE LEAF intervention to have a high level of feasibility and acceptability. In general, participants liked the social features that were implemented and expressed feedback about how they could be improved to enhance their usability and foster a sense of social connection. Future iterations of the SAGE LEAF intervention may include a more extensive onboarding process describing how the social features work and how they may benefit caregivers. We hope that future refinements to the intervention will enhance the perception that there are other caregiver participants and study staff involved, thereby fostering great engagement among participants.

## Abbreviations

SAGE LEAF: Social Augmentation of Self-Guided Electronic Delivery of the Life Enhancing Activities for Family Caregivers
